# Construction and validation of a prediction model for 90-day readmission risk in patients with chronic heart failure

**DOI:** 10.3389/fcvm.2025.1627789

**Published:** 2025-11-06

**Authors:** Qianqian He, Ze Lai, Yangkai Shi, Beibei Zou, Chao Feng

**Affiliations:** 1 Department of Cardiology, The Fourth Affiliated Hospital of School of Medicine, and International School of Medicine, International Institutes of Medicine, Zhejiang University, Yiwu, China; 2 Liangzhu Laboratory, Zhejiang University Medical Center, Hangzhou, China

**Keywords:** heart failure, prediction model, readmission, LASSO regression, nomogram

## Abstract

**Background:**

Chronic heart failure (CHF) is associated with high morbidity and mortality rates, which is not curable currently, resulting in an increasing risk of readmission and imposing a considerable burden on healthcare systems. Predictive modeling is a critical tool for guiding the clinical management of CHF. 90-day is a crucial time point for readmission risk assessment in patients with CHF. However, there is a lack of risk factor exploration, as well as predictive modeling for 90-day readmission risk in these patients. The aim of this study is to identify prognostic risk biomarkers and develop a novel prediction model for 90-day readmission for patients with CHF.

**Methods:**

542 CHF patients hospitalized at the Department of Cardiology, the Fourth Affiliated Hospital of Zhejiang University were randomly split into training (*N* = 380) and validation (*N* = 162) cohort at a 7:3 ratio. Demographic, comorbidities, laboratory tests, and echocardiography results were analyzed through Least Absolute Shrinkage and Selection Operator (LASSO) regression to select predictive variables. Furthermore, receiver operating characteristic (ROC) curve, the area under the curve (AUC), decision curve analysis (DCA), and calibration curves were used to access the discriminative power, clinical validities, and calibration of the model.

**Results:**

Of the included 542 patients, the readmission rates were 18.7% and 19.1% in 90-day follow-up in the training and validation cohort respectively. Five variables, including cardiac troponin (cTn), fasting blood glucose (FBG), serum sodium, estimated glomerular filtration rate (eGFR), neutrophil (NEU) showed the strongest correlation with 90-day readmission according to LASSO regression. These selected variables were then combined into a novel prediction model, with an AUC of 0.746 [95% (confidence interval) CI: 0.685–0.808] in the training cohort and 0.705 (95% CI: 0.605–0.804) in the validation cohort.

**Conclusions:**

Our findings suggest that a predictive model incorporating the variables of cTn, FBG, serum sodium, eGFR and NEU demonstrating a good predictive ability for 90-day readmission risk in patients with CHF, which can aid clinicians in clinical decisions and personalized management.

## Introduction

Heart failure (HF) is a profoundly intricate and perilous syndrome, distinguished by substantial morbidity and mortality, significantly compromised functional capacity and quality of life, thus becoming public concern and imposing a considerable burden on global healthcare systems (1). Despite notable advancements in interventions for HF, it is estimated that 64 million people are affected by HF worldwide, with its prevalence rising significantly in numerous middle- and low-income nations (2, 3). Chronic heart failure (CHF) represents the predominant clinical entity within the HF spectrum, accounting for the majority of cases in clinical practice (4). And the primary causes of CHF include ischemic heart disease, hypertension, dilated cardiomyopathy, rheumatic valve disease, and non-rheumatic valve heart disease (5), all of which are common diseases in the elder generation along with an aging population.

CHF stands as a predominant etiology of admissions among adult and geriatric populations (1), characterized by its multifactorial causes, persistent progression, and limited therapeutic options, is associated with substantially high readmission rates (6). Approximately 25% CHF patients are readmitted within 30 days after discharge, and nearly 50% experience readmission within 6 months (7, 8). It is estimated that up to 25% HF readmissions could potentially be prevented (8). Therefore, early detection of readmission risks for patients with CHF and implementation of timely intervention are crucial for enhancing clinical prognosis for patients (9).

While conventional predictive models for CHF readmission have been developed, with extended follow-ups and limited variable selection (10, 11). Over recent years, there are multiple 30-day readmission risk-prediction models for patients with CHF, which aim to focus on 30-day outcomes (12, 13). Nevertheless, window of vulnerability is expected to be longer, the peak in the incidence rate of event occurs within 90 days post-discharge, after which it tends to stabilize (14, 15). Thus, it is imperative to construct a novel prediction model to access the risk of readmission for CHF patients in a period of 90-day. Early identification of CHF risk factors and timely therapeutic intervention contribute to diminish the risk of readmission and help to alleviate the financial burden on families and societies.

In order to fill the research gaps mentioned above, we established a clinical cohort dataset through the retrospective collection of hospitalization data from patients diagnosed with CHF. And we aimed to establish and validate a 90-day readmission nomogram for CHF patients. It would be helpful in identifying potential risks, facilitating prompt clinical decision-making, and supporting basic research into the mechanisms of CHF.

## Methods

### Study design and source of data

Data of CHF patients admitted at the Department of Cardiology, the Fourth Affiliated Hospital of Zhejiang University, were retrospectively collected between October 2014 and December 2023 to establish a clinical cohort dataset. The inclusion criteria were as follows: (1) CHF diagnosed based on the 2021 ESC Guidelines for the diagnosis and treatment of acute and chronic heart failure (4), and patients were diagnosed by two experienced cardiologists; (2) New York Heart Association (NYHA) cardiac function class II-IV; (3) age >18 years. The exclusion criteria were as follows: (1) patients lost to follow-up; (2) missing key clinical data; (3) history of malignant tumors; (4) severe end-stage diseases of vital organ such as the liver, kidney or brain. Existing evidence suggests substantial overlap in predictive factors between HF with preserved ejection fraction (HFpEF) and reduced ejection fraction (HFrEF), thus they are all included in this study (16). The primary endpoint of this study was unplanned HF readmission within 90-day after first readmission for patients with CHF. Patients were followed up for 90 days after discharge from the index admission. To ensure accurate tracking, we conducted telephone interviews and further verified their clinical status using hospital records.

A total of 542 patients with CHF were ultimately included in our study and we defined it as the whole cohort dataset. The dataset was randomly split into a training set and a validation set using the sample function in R, with a fixed random seed to ensure reproducibility, we divided the whole cohort set into training and validation set at a ratio of 7:3, with 380 patients in the training cohort and 162 patients in the validation cohort. This study was reviewed and approved by the Institutional Ethical Committee of the Fourth Affiliated Hospital, Zhejiang University School of Medicine (approval number: K2025056). All procedures were conducted in accordance with the Declaration of Helsinki, as well as the relevant guidelines and regulations of the Institutional Ethical Committee, and the informed consent was waived due to the retrospective design.

### Data collection

The data for this retrospective study were obtained from the electronic medical records (EMRs) of the Fourth Affiliated Hospital of Zhejiang University for patients at the time of first hospitalization, covering the period from October 2014 to December 2023. Furthermore, we conducted a follow-up to monitor the readmission status of patients within a 90-day period post-discharge. Data collection encompassed demographic information such as sex, age, smoking, drinking, comorbidities such as diabetes, valve heart disease, atrial fibrillation (AF), myocardial infarction (MI), coronary artery disease (CAD), cerebrovascular disease and chronic obstructive pulmonary disease (COPD), clinical characteristics such as body mass index (BMI, kg/m^2^), systolic (SBP, mmHg) and diastolic (DBP, mmHg) blood pressure, admission heart rate (HR, bpm), laboratory test results such as fasting blood glucose (FBG, mmol/L), total cholesterol (TC, mmol/L), triglycerides (TG, mmol/L), high-density lipoprotein (HDL, mmol/L), low-density lipoprotein (LDL, mmol/L), total bilirubin (TBIL, μmol/L), creatine kinase MB (CK-MB, U/L), alanine aminotransferase (ALT, U/L), aspartate aminotransferase (AST, U/L), serum sodium (mmol/L), estimated glomerular filtration rate (eGFR, mL/min/1.73 m^2^), Brain Natriuretic Peptide (BNP, ng/L), cardiac troponin (cTn, ng/L), neutrophil (NEU, %), monocyte (MON, %), hemoglobin (g/L), platelet (PLT, 10^9^/L), echocardiography results such as left ventricular ejection fraction (LVEF, %) and left atrium diameter (LAD, mm), and a novel clinical biomarker, CALLY index (17–19). BMI was calculated by dividing weight in kilograms by the square of height in meters. The CALLY index was calculated as follows: albumin (g/dL) × lymphocyte (cells/μL)/CRP (mg/dL) × 10^4^ (19).

### Statistical analysis

In this study, statistical analyses were performed using R (version 4.4.2) and SPSS (version 27.0). Continuous data following a normal distribution were presented as mean ± standard deviation (mean ± SD), and intergroup comparisons were conducted using the independent samples t-test. For data that followed a non-normal distribution, interquartile ranges (IQR; P25, P75) were used for representation, and nonparametric tests were applied for intergroup comparisons. Normality was assessed using the Shapiro–Wilk test. Categorical data were expressed as frequencies and percentages (%), and intergroup comparisons were performed using the chi-square test. All tests were two-sided and *P*-value < 0.05 was considered statistically significant. Pairwise correlations and VIFs were calculated, applying a pre-specified exclusion threshold (VIF >2.5 |*r*| > 0.7). The Least Absolute Shrinkage and Selection Operator (LASSO) regression analysis was employed to reduce data dimensionality and identify the most significant variables. Subsequently, the prediction model was constructed by incorporating the variables selected through LASSO Regression. The discriminative ability of the model was evaluated using the area under the receiver operating characteristic curve (AUC), and calibration curves accompanied by the Homster-Lemeshow test were used to assess model accuracy. Decision curve analysis (DCA) was applied to quantify the net clinical benefit and validate the practical utility of the nomogram. Internal validation of the model was performed using the validation data set. To enhance the clinical applicability of our prognostic model, we performed risk stratification based on the computed risk scores. The optimal cutoff value for distinguishing between high-risk and low-risk groups was determined using the Youden index. The threshold that maximized the Youden index was selected as the optimal cutoff point. This empirically derived cutoff value was then applied to categorize patients in both the training and validation cohorts into high-risk and low-risk groups. The discriminative ability of this stratification was evaluated using Kaplan–Meier survival analysis with log-rank tests for time-to-event outcomes, and by comparing 90-day readmission rates between risk groups using chi-square tests for binary outcomes (20).

## Results

### Clinical characteristics of the study cohort

**Figure 1:**
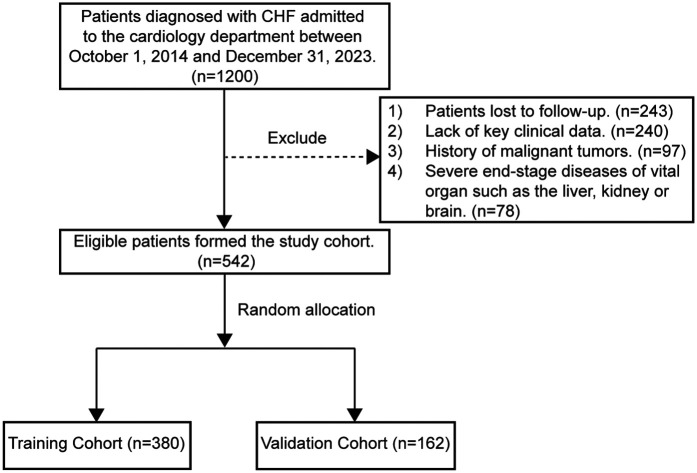
Flow chart of inclusion and exclusion criteria in patients admitted with chronic heart failure. CHF, chronic heart failure.

The study flowchart is depicted in Figure 1. A total of 1,200 CHF patients were screened to participate in this study. Among them, 658 cases were excluded and 542 cases remained in the final study, in which 380 patients and 162 patients were in the training and validation cohort respectively (Table 1). There were 71 (18.7%) and 31 (19.1%) CHF patients readmitted to the hospital within 90-day follow-up in the training and validation cohort. Table 1 summarizes the demographic and clinical characteristics of the two cohorts. Baseline characteristics were generally comparable between the training and validation cohorts. To select the initial variables, we made a collinearity diagnosis of candidate variables at baseline and excluded TC, TG, HDL and LDL for further analysis, with which VIF >2.5 (Supplementary Table S1) or |*r*| > 0.7 (Supplementary Table S2).

**Table 1:** Baseline characteristics of patients with chronic heart failure in the training and validation cohort.

Variables	Training cohort (*n* = 380)	Validation cohort (*n* = 162)	*p*-value
Demographics			
Male, *n* (%)	213 (56.1)	100 (61.7)	0.259
Age, y	71.00 [63.00, 78.00]	70.00 [62.00, 78.00]	0.512
Smoking, *n* (%)	95 (25.0)	44 (27.2)	0.675
Drinking, *n* (%)	71 (18.7)	33 (20.4)	0.736
Comorbidities			
Diabetes, *n* (%)	94 (24.7)	40 (24.7)	1.000
Valve heart disease, *n* (%)	44 (11.6)	17 (10.5)	0.828
Atrial fibrillation, *n* (%)	112 (29.5)	43 (26.5)	0.557
Myocardial infarction, *n* (%)	40 (10.5)	16 (9.9)	0.942
Coronary artery disease, *n* (%)	218 (57.4)	107 (66.0)	0.073
Cerebrovascular disease, *n* (%)	51 (13.4)	19 (11.7)	0.691
COPD, *n* (%)	74 (19.5)	37 (22.8)	0.440
Clinical characteristics			
BMI, kg/m^2^	24.61 [21.82, 26.90]	23.96 [21.48, 26.88]	0.657
SBP, mmHg	135.00 [119.00, 150.00]	129.00 [118.25, 144.00]	0.027
DBP, mmHg	76.00 [68.00, 85.00]	74.00 [66.00, 84.00]	0.137
Admission heart rate, bpm	80.00 [71.00, 93.00]	76.00 [68.00, 87.00]	0.048
Laboratory test			
FBG, mmol/L	5.01 [4.46, 5.95]	5.00 [4.57, 5.90]	0.664
TC, mmol/L	3.55 [2.89, 4.28]	3.54 [2.99, 4.15]	0.873
TG, mmol/L	1.17 [0.90, 1.65]	1.12 [0.85, 1.45]	0.123
HDL, mmol/L	1.07 [0.89, 1.26]	1.08 [0.91, 1.27]	0.717
LDL, mmol/L	1.83 [1.37, 2.40]	1.78 [1.45, 2.41]	0.778
TBIL, μmol/L	11.40 [8.07, 15.70]	11.45 [8.62, 16.60]	0.329
CK-MB, U/L	12.90 [10.00, 16.92]	12.75 [10.00, 16.85]	0.907
ALT, U/L	17.00 [12.00, 29.00]	19.00 [13.25, 27.00]	0.422
AST, U/L	24.00 [19.00, 32.00]	24.00 [19.00, 32.00]	0.884
Serum sodium, mmol/L	140.82 [138.80, 142.20]	140.55 [138.65, 142.28]	0.969
e-GFR, mL/min/1.73 m^2^	73.00 [47.75, 93.00]	81.00 [59.25, 98.75]	0.003
BNP, *n* (%)	314 (82.6)	131 (80.9)	0.712
cTn, *n* (%)	205 (53.9)	76 (46.9)	0.160
NEU, %	65.50 [57.58, 72.43]	65.10 [57.90, 74.38]	0.464
MON, %	8.25 [6.70, 10.00]	8.20 [6.90, 10.30]	0.508
Hemoglobin, g/L	334.00 [328.00, 341.00]	334.00 [327.00, 341.75]	0.967
PLT, 10^9^/L	180.50 [142.75, 218.00]	184.50 [145.25, 227.75]	0.574
CALLY index	2.00 [0.30, 6.60]	1.40 [0.32, 5.50]	0.392
Echocardiography results			
LVEF, %	60.00 [47.32, 66.50]	62.05 [47.00, 67.95]	0.182
LAD, mm	38.85 [33.00, 43.00]	37.00 [32.02, 42.00]	0.084
First readmission within 90-day, *n* (%)	71 (18.7)	31 (19.1)	0.998

COPD, chronic obstructive pulmonary disease; BMI, body mass index; SBP, systolic blood pressure; DBP, diastolic blood pressure; FBG, fasting blood glucose; TC, total cholesterol; TG, triglycerides; HDL, high-density lipoprotein; LDL, low-density lipoprotein; TBIL, total bilirubin; CK-MB, creatine kinase MB; ALT, alanine aminotransferase; AST, aspartate aminotransferase; e-GFR, estimated glomerular filtration rate; BNP, Brain Natriuretic Peptide; cTn, cardiac troponin; NEU, neutrophil; MON, monocyte; PLT, platelet; CALLY index, C-reactive protein-albumin-lymphocyte index; LVEF, left ventricular ejection fraction; LAD, left atrium diameter.

### Selection of risk variables and derivation of the prediction model

**Figure 2:**
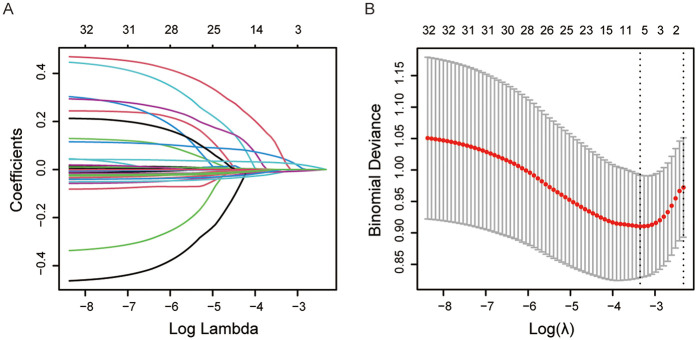
The LASSO regression analysis identified variables correlated with 90-day readmission risk in CHF patents. **(A)** Number of non-zero coefficients in the model. **(B)** Number of variables corresponding to different *λ* values.

**Figure 3:**
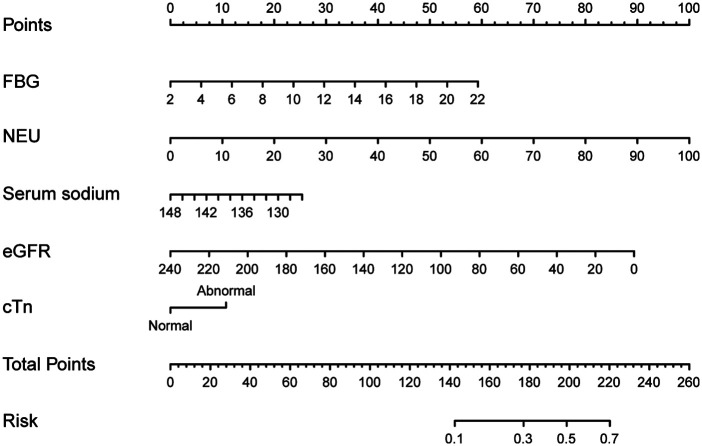
Nomogram for 90-day readmission in patients with chronic heart failure. FBG, fasting blood glucose; NEU, neutrophil; e-GFR, estimated glomerular filtration rate; cTn, cardiac troponin.

Using LASSO regression with 10-fold cross-validation in the training set, the lambda_min was determined to be 0.03504438, and lambda_1se was identified as 0.09751307 (Figure 2). Of the 31 variables examined, 5 non-zero coefficients were confirmed as significant variables, including FBG, NEU, serum sodium, eGFR and cTn. The predictive model for 90-day readmission in CHF patients was then developed incorporating the 5 variables selected by LASSO regression and depicted as a nomogram (Figure 3).

### Evaluation and validation of the nomogram performance

**Figure 4:**
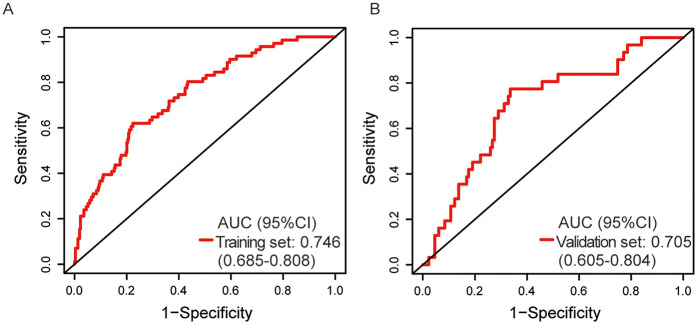
Receiver operating characteristic curve of prediction model for patients with chronic heart failure in the training **(A)** and validation **(B)** cohort. AUC, area under the curve; CI, confidence interval.

**Figure 5:**
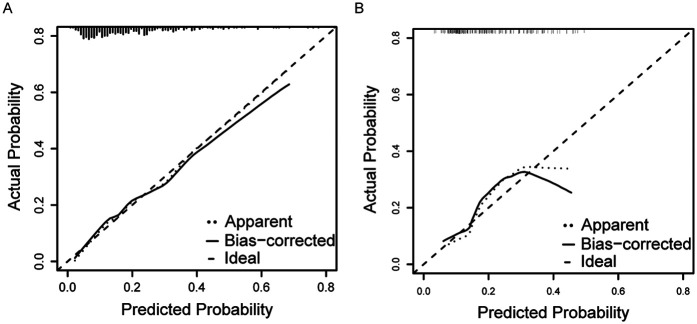
Calibration curve of prediction model for patients with chronic heart failure in the training **(A)** and validation **(B)** cohort.

**Figure 6:**
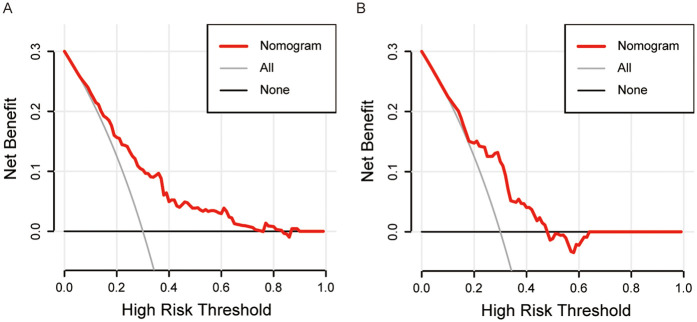
Decision curve analysis for patients with chronic heart failure in the training **(A)** and validation **(B)** cohort.

The ROC curve analysis for the nomogram yielded an AUC of 0.746 [95% (confidence interval) CI: 0.685–0.808] and 0.705 (95% CI: 0.605–0.804) in the training cohort and validation cohort respectively (Figure 4), which suggests the reliable discriminative ability of our nomogram. Then we applied calibration curve which utilized bootstrapping with 1,000 resamples to confirm the precision of the nomogram in both cohorts (Figure 5). The Hosmer-Lemeshow goodness of fit test showed a *p*-value of 0.213 for the training set and of 0.195 for the validation set, indicating the model was moderately calibrated. In order to verify the clinical application of this nomogram, DCA curves were drawn for both cohorts (Figure 6), suggesting that the nomogram could have good benefits in clinical practice.

### Risk stratification

**Figure 7:**
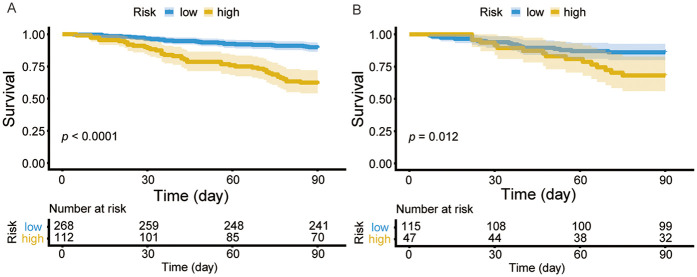
Kaplan–meier curve analysis between high- and low- risk CHF patients in the training cohort **(A)** and validation cohort **(B)**.

Using the Youden index, an optimal cutoff value of 0.232 was identified from the training cohort to stratify patients into high-risk and low-risk groups in training and validation cohorts (Figure 7), with between-group differences assessed by log-rank test in both cohorts (*P* < 0.05). The chi-square test demonstrated a significant difference in 90-day readmission risk between the high-risk and low-risk groups in both the training (*χ*^2^ = 41.6, *P* < 0.001) and validation cohorts (*χ*^2^ = 5.872, *P* < 0.001).

## Discussion

In the current study, it is the first time to develop and validate an easy-to-use and relatively personalized model for predicting 90-day readmission risk for patients with CHF, which helps optimize clinical management. Risk prognostic factors were retrospectively identified through LASSO regression and a simple prediction nomogram was developed and validated in this study. This nomogram integrated five independent risk factors, including FBG, NEU, serum sodium, eGFR and cTn, which yielded an AUC of 0.746. The validation cohort demonstrated an AUC of 0.705. With this nomogram, clinicians can comprehensively calculate the risk scores of CHF patients within the critical 90-day time point, effectively screening CHF patients who are at high risk of readmission and thus making timely clinical decisions and implementing early targeted intervention, which can lead to improved clinical outcomes.

Despite current therapeutic strategies, patients hospitalized for HF face substantial risks during the early post-discharge period, with mortality and readmission rates reaching 15% and 30% respectively within 60–90 days. This high-risk interval, clinically designated as the vulnerable phase, contributes disproportionately to the >US$30 billion annual economic burden of HF care in the United States (15). The vulnerable phase is defined as the immediate post-discharge period. While adverse clinical outcomes including morbidity and mortality are observed during hospitalization, most adverse events emerge in HF patients during post-discharge period (21). The exact length of the post-discharge vulnerable phase remains unclear, limited evidence suggests a 60–90 days high-risk period (14, 22). In the EVEREST trial, 32% patients were readmitted for cardiovascular causes during the 90-day post-discharge period (22). Thus, compared with previous HF readmission models, we considered that the 90-day readmission are more in line with the clinical practice. Nevertheless, the risk factors and prediction models targeting 90-day vulnerable phase remain uninvestigated.

The model we constructed covers biomarkers for assessing multiple aspects of CHF, which are also easily accessible in the clinical practice. cTn is a significant cardiac biomarker for HF patients and it is widely utilized in the management of HF patients, with latest guidelines strongly endorsing its application for diagnosis, risk stratification, and monitoring disease progression (23). Elevated levels of cTn were detected in the majority of patients with acute or chronic HF and it shows a significant predictive ability of mortality in HF patients (24, 25). In a meta-analysis on patients with CHF, cTn was verified as an independent risk factor of all-cause death, cardiovascular death and readmission (26). It is recommended in ACC/AHA guidelines that monitoring cTn levels at the time of hospital admission to facilitate risk stratification (27). Compared with CK-MB, and myoglobin, it has been proved that cTn is a more sensitive and specific biomarker in diagnosis and prognosis prediction for cardiomyocyte injury (28, 29).

In the pathophysiological cascade of CHF, it is widely believed that inflammation function exerts pivotal influences (30), and its involvement in the initiation and progression of HF has long been observed (31). NEU have been regarded as primary inflammatory biomarker in cardiovascular diseases (CVD). In cardiovascular inflammation, NEU promotes repair via endothelial regeneration and angiogenesis, while in cardiac hypertrophy and stroke, it exacerbates damage by activating macrophages and enhancing coagulation (32). It significantly contributes to the mediation of tissue injury and cardiac remodeling in the deterioration of CVD, which is associated with the severity of HF and overall mortality in patients with different HF etiologies (33, 34).

Clinical indicators such as serum sodium, eGFR and FBG delineated the stress state associated with acute exacerbation of HF. Hyponatremia, characterized by a serum sodium concentration below 135 mEq/L, predominantly arises from water retention. Even slight hyponatremia is linked to prolonged hospitalization and elevated mortality rates (35). Hyponatremia and lower serum sodium levels are associated with elevated all-cause mortality in patients with HF (36). Our study also showed that the decreased serum sodium increased the risk of readmission in patients with CHF. Renal dysfunction commonly occurs in advanced HF, especially in severe cases, which is generally defined as cardiorenal syndrome (CRS).CRS is a complicated group of disorders involving dysfunction in both the heart and kidneys (37). Over 60% patients admitted for HF also exhibit concurrent CKD with an eGFR of <60 ml/min per 1.73 m^2^ (38). Renal dysfunction in acute decompensated CHF may result from elevated intra-abdominal pressure, increased central venous pressure, and renal venous congestion (39). The existence of CKD significantly elevates the risk of adverse outcomes, particularly in individuals with lower baseline eGFR levels (40), which is also in accordance with our results. It is suggested that prompt intervention for renal dysfunction may mitigate adverse events in HF patients (41). A population-based cohort have found that there was a continuous, independent, and positive correlation between FBG and HF, with the hazard ratio (HR) for HF per 1 mmol/L increase in FBG was 1.34 (95% confidence interval 1.22–1.48) (42). Several mechanisms that a high level of FBG adversely damages cardiac function through the accumulation of advanced glycosylation end-products (AGEs), activation of inflammation and oxidative stress, have been proposed to explain the causal relationship between elevated FBG and the pathogenesis of HF (43).

Our study is dedicated to screen candidate clinical biomarkers which have not been extensively investigated in CVD and then construct a novel prediction model to predict the 90-day readmission risk for CHF patients, and the selected risk factors were also broadly supported by relevant studies. However, there are still several limitations in this study. Firstly, the patients were from the Cardiology Department in a single-center population, it may not be generalized to other centers or the severe HF patients admitted to the emergency department or intensive care unit. Secondly, the generalizability of our study may be limited by the relatively small sample size, thus a larger cohort would be required to validate our results. Thirdly, this retrospective analysis is inherently vulnerable to confounding factors and potential deficiencies in data recording, so it is in urgent need to conduct a prospective study to confirm the robustness. Finally, we acknowledge that the associative nature of our analysis limits definitive causal interpretations. As pioneeringly applied in other clinical domains (44–46), a logical and valuable extension of this work would be to construct a structural equation model (SEM), which can disentangle direct, indirect, and mediating effects among clinical variables.

This article lays the foundation for later construction of models related to the risk of readmission as well as adverse events in CHF patients within 90-day. From our perspective, some emerging clinical scores, like the CHA2DS2-VASc score, may appear more frequently in future studies and further improve the accuracy of prognostic prediction for CHF patients. These scores or metrics are usually assessed and calculated for multiple aspects of the patient, and there have been a number of studies that have shown promise in their application (47, 48).

## Conclusions

In conclusion, CHF patients with abnormal cTn levels, lower serum sodium level, higher FBG levels, lower eGFR levels and higher NEU levels were associated with higher risk of 90-day readmission. The nomogram obtained in this study is reliable and accessible in clinical practice, which provides a simple and graphical interface that clinicians can utilize to quickly evaluate prognosis and help prompt clinical decision-making. Meanwhile, our predictive model enriches the short-term CHF readmission model represented by 90-day, which helps the development of personalized service. Further prospective and multi-centered research will be welcomed to optimize this model.

## Data Availability

The raw data supporting the conclusions of this article are available from the corresponding author on reasonable request.

## Supplementary material

Supplementary Table 1 Variance inflation factors for variables.

Supplementary Table 2 Correlation matrix of variables.
